# Osteoblast-like Cell Differentiation on 3D-Printed Scaffolds Using Various Concentrations of Tetra-Polymers

**DOI:** 10.3390/biomimetics7020070

**Published:** 2022-05-31

**Authors:** Nattanan Wattanaanek, Srisurang Suttapreyasri, Bancha Samruajbenjakun

**Affiliations:** 1 Orthodontic Section, Department of Preventive Dentistry, Faculty of Dentistry, Prince of Songkla University, Hat Yai 90112, Songkhla, Thailand; wa_n30@hotmail.com; 2 Sirindhorn College of Public Health Khon Kaen, Khon Kaen 40000, Khon Kaen, Thailand; 3 Department of Oral and Maxillofacial Surgery, Faculty of Dentistry, Prince of Songkla University, Hat Yai 90112, Songkhla, Thailand; srisurang.s@psu.ac.th

**Keywords:** ion-deposited, 3D-printed scaffold, amorphous calcium phosphate, calcium sulfate hemihydrate, osteoblast-like cell differentiation

## Abstract

New bone formation starts from the initial reaction between a scaffold surface and the extracellular matrix. This research aimed to evaluate the effects of various amounts of calcium, phosphate, sodium, sulfur, and chloride ions on osteoblast-like cell differentiation using tetra-polymers of amorphous calcium phosphate (ACP), calcium sulfate hemihydrate (CSH), alginic acid, and hydroxypropyl methylcellulose. Moreover, 3D-printed scaffolds were fabricated to determine the ion distribution and cell differentiation. Various proportions of ACP/CSH were prepared in ratios of 0%, 13%, 15%, 18%, 20%, and 23%. SEM was used to observe the morphology, cell spreading, and ion complements. The scaffolds were also examined for calcium ion release. The mouse osteoblast-like cell line MC3T3-E1 was cultured to monitor the osteogenic differentiation, alkaline phosphatase (ALP) activity, total protein synthesis, osteocalcin expression (OCN), and calcium deposition. All 3D-printed scaffolds exhibited staggered filaments, except for the 0% group. The amounts of calcium, phosphate, sodium, and sulfur ions increased as the amounts of ACP/CSH increased. The 18%ACP/CSH group significantly exhibited the most ALP on days 7, 14, and 21, and the most OCN on days 14 and 21. Moreover, calcium deposition and mineralization showed the highest peak after 7 days. In conclusion, the 18%ACP/CSH group is capable of promoting osteoblast-like cell differentiation on 3D-printed scaffolds.

## 1. Introduction

The interaction between the scaffold surface and the composition of the extracellular matrix (ECM) plays a pivotal role in bone regeneration. The deposition of chemical ions on the scaffold surface demonstrates a bioactive environment initiation, which effects cell attachment, proliferation, and mineralization [1]. Within two months, a calcium-phosphate (Ca-P)-rich layer on an implant material composed of MgO, CaO, SiO_2_, P_2_O_5_, and CaF_2_ can release ion elements that directly react with carbonate compounds in the body fluid to form small grains of crystalline hydroxyapatite [2].

Three-dimensional (3D) computer-aided design makes it easy to control the interconnected structure of the scaffold and support the nutrient-supplied channels for cell growth in the later stages [3,4]. Previous research demonstrated that 3D-printed scaffolds had considerably higher markers of osteoblast cell differentiation, alkaline phosphatase (ALP) activity, and osteonectin and osteocalcin expression than cultures in 2D scaffolds in various time periods. Hence, 3D scaffolds might provide a greater ability for cells to deposit Ca-P ions than 2D cultures [5].

Ca-P and calcium sulfate are used most often as inorganic materials for bone substitutes [6,7]. Amorphous calcium phosphate (ACP) is a kind of calcium phosphate bioceramic [8,9], which is a poor inorganic ECM. Ca-P released from ACP has been shown to induce initial precipitation of ECM components to form hydroxyapatite [10]. Calcium sulfate hemihydrate (CSH) is a popular biocompatible material that is often added to Ca-P groups. For example, it can be combined with β-tricalcium phosphate as an injectable material for widespread use in various defects that need tissue engineering integration [11,12,13]. Moreover, the combination of CSH and ACP is interesting because of the Ca-P-rich surface and structural stability of a graft in a bony defect area. The bi-inorganic composition showed new bone formation in rats within three months and could gradually resorb from the start of new bone formation [14].

Alginic acid (Alg) is an excellent option as an organic polymer for use in tissue engineering because it can form a robust hydrogel, and it is biocompatible and biodegradable [15,16]. In addition, it is a natural biocompatible polymer, used mostly as a base with other materials for fabricating scaffolds because filament stability can be controlled during 3D printing. Furthermore, the biological properties showed cell progression and differentiation [17,18]. Hydroxypropyl methyl cellulose (HPMC) is a polysaccharide that improves the properties for 3D printing due to its long-term solubility as a natural polymer. A previous study found that HPMC is a biosafe polymer that could promote cell viability and growth [19]. Moreover, two polymers used to form an interpenetrated polymer network enhanced the mechanical properties of the gel compared to single-network systems [20,21].

The 3D structure and various amounts of Ca-P from both ACP and CSH were mimicked as ECM that might affect osteoblast cell differentiation. This study aimed to evaluate the effect of ion elements from tetra-polymers using 3D-printed scaffolds on mouse osteoblast-like cell differentiation.

## 2. Materials and Methods

### 2.1. Composite Gel Preparation

ACP powder was made by combining 2.33 M sodium phosphate dibasic solution (Sigma-Aldrich, St. Louis, MO, USA) and a 3.50 M calcium chloride (CaCl_2_) solution (Sigma-Aldrich) in sodium hydroxide, and then dissolving it in distilled water. CSH was obtained from Sigma-Aldrich in Germany.

At 120 °C, 1 wt% Alg (Sigma-Aldrich) and 0.9% HPMC (Sigma-Aldrich) were dissolved in phosphate-buffered saline (PBS). ACP and CSH were added at a ratio of 2:3 to the gel in various amounts, which resulted in 13%ACP/CSH, 15%ACP/CSH, 18%ACP/CSH, 20%ACP/CSH, and 23%ACP/CSH. A 0%ACP/CSH group served as the control. To achieve a homogeneous and desirable particle size, the printing ink dispersion was filtered through a 110 μm filter [22].

### 2.2. Scaffold Preparation

**Figure 1:**
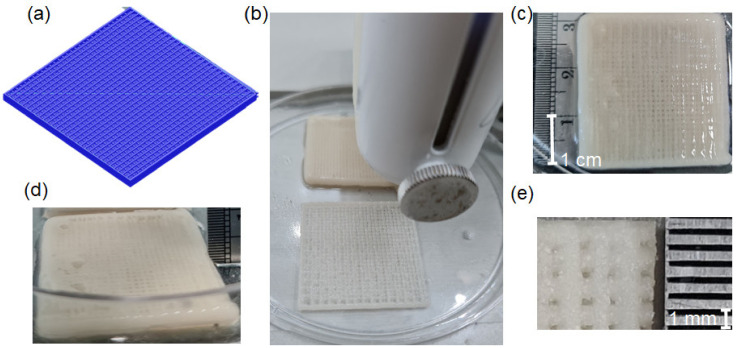
(**a**) Simulation from the G-code-designed 3D scaffold. (**b**) Pneumatic head for the extrusion of each filament. (**c**) Top view of the completed 3D-printed scaffold (30 × 30 × 2 mm). (**d**) Cross-sectional view of the scaffold. (**e**) Freeze-dried scaffold after cutting into 7 × 7 × 2 mm sections.

G-code generating software was used to create the staggered scaffold (Repetier-Host V2.1.6, Willich, Germany). This structure had 30 filaments per layer at 0° and 90° angles in each layer until 10 layers were fabricated. The filaments were separated by 1 mm. After a 1-day wait for the gel to develop (Figure 1a), the gel was transferred to a 3 mL syringe and extruded using a nozzle tip with a diameter of 0.02 mm. The pneumatic 3D-printer (Bio X™, Cellink, Blacksburg, VA, USA) was programmed at 45 mm/s, 350 kPa, and 25.0 °C print head temperature. At every 2–3 layers, 0.1 mM CaCl_2_ was sprayed as a crosslink for the permanent structure (Figure 1b). The scaffold was immediately immersed in crosslink for 60 min to complete the set, and then cleaned 5 times with distilled water before freeze-drying for 3 h. Each scaffold was divided into 7 × 7 × 2 mm sections and disinfected using ethylene oxide gas before seeding the cells. All groups were divided into three samples each (Figure 1c–e) [22].

### 2.3. Cell Adhesion

Scanning electron microscopy (SEM) (Quanta 400, Thermo Fischer Scientific, Brno, Czech Republic) was used to observe the characterized osteoblast cell adhesion on the surface of the scaffold after gold coating at 15 kV.

### 2.4. Ion Complements

SEM and energy-dispersive spectroscopy (EDS) (Quanta 400, Thermo Fischer Scientific, Brno, Czech Republic) were used to observe the percentages of carbon (C), oxygen (O), sodium (Na), calcium (Ca), phosphate (P), sulfur (S), and chloride (Cl) ions of 100% of the sample surfaces selected at random and repeated three times. The EDS-SEM map represented the distribution of each ion.

### 2.5. Calcium Release Analysis

The scaffolds were soaked in 1X PBS on days 1, 3, 7, 14, and 21. The solution was collected and tested with a calcium colorimetric assay kit (BioVision Ins., Milpitas, CA, USA) at OD 575 nm.

### 2.6. Cell Culture

The mouse osteoblast cell line MG3T3-E1 (ATCC, Manassas, VA, USA) was cultured with alpha-MEM medium (α-MEM, Gibco™, Invitrogen, Carlsbad, CA, USA), 10% fetal bovine serum, 1% penicillin/streptomycin, and 0.1% Fungizone^®^. The sterilized scaffolds were placed into a 48-well plate and cell seeding was conducted at 1 × 10^5^ cells/well. The medium was changed every 3 days with 0.5 L/well. Dexamethasone (500 M) (Sigma-Aldrich^®^), 0.005 g/mL ascorbic acid (Gibco™), and 1 M β-glycerophosphate (Sigma-Aldrich, St. Louis, MO, USA) were used to produce the osteogenic medium. After three days, the cultivated cells were switched to osteogenic media to begin cell differentiation. After the cells were cultivated for 3, 7, 14, and 21 days, the scaffolds were washed twice in 1X PBS and then soaked in 1% Triton X-100 in PBS. Freeze-thawing was used to remove cellular protein by freezing the scaffold at −80 °C for 1 h, and then returning it to room temperature for 1 h. The freeze-thaw procedure was repeated three times. The scaffolds were crushed and centrifuged at 12,000 RPM for 10 min. A clear solution was collected for cell lysis investigation.

### 2.7. Alkaline Phosphatase (ALP) Activity

The early stage of cell differentiation was detected by the alkaline phosphatase liquicolor kit (Wiesbaden, Germany) on days 3, 7, 14, and 21. The clear lysate solution of a 20 μL sample was mixed with 1000 μL of AMP buffer at 37 °C for a 1-min incubation. An amount of 250 μL of the substrate (60 mmol/L of p-nitrophenyl phosphate and 0.095% of sodium azide) was then added. The activity was measured photometrically (OD 420 nm).

### 2.8. Total Protein Synthesis

The total amount of protein synthesized was measured using the Bio-Rad protein assay dye reagent (Bio-Rad Laboratories Inc., Hrcules, CA, USA) concentrate using the bicinchoninic acid method under an absorbance of 562 nm on days 7, 14, and 21. The amount of protein was determined from a standard curve.

### 2.9. Osteocalcin (OCN) Analysis

The mouse OC/BGP (osteocalcin) production on the scaffolds was measured using an ELISA kit (Elabscience^®^, Houston, TX, USA) to analyze late cell differentiation by detecting the sandwich antibody-antigen complex in the lysate solution following the instructions on days 14 and 21.

### 2.10. Calcium Content

The lysis solutions on days 7, 14, and 21 were used to detect calcium using a calcium colorimetric assay kit (BioVision Inc., Milpitas, CA, USA).

### 2.11. Mineralization Analysis

Alizarin red dye (Sigma-Aldrich, St. Louis, MO, USA) was used to identify mineralization deposition. All scaffolds were washed twice with 1X PBS, and the cells were fixed with 4% formaldehyde. An amount of 1 mL of alizarin red solution was dropped onto the scaffold at room temperature. After 20 min, the scaffold was rinsed with distilled water and observed under light microscopy on days 14 and 21.

### 2.12. Statistical Analysis

The mean and standard deviation of all measurements were calculated. The distribution was tested with the Shapiro–Wilk test and all of the data had a normal distribution. One-way ANOVA and Tukey’s HSD test (SPSS Statistics Bass 17.0 for Windows EDU, Chicago, IL, USA) were used to compare the groups. The power of the test was 0.8. A p-value of 0.05 was used to determine statistical significance. 

## 3. Results

### 3.1. Scaffold Morphology and Cell Attachment

**Figure 2:**
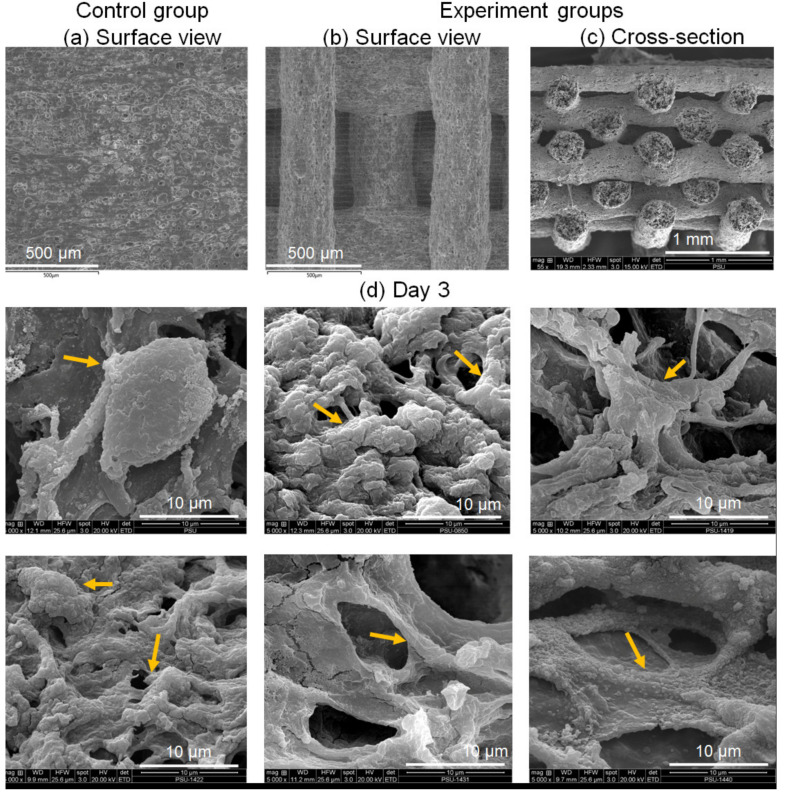
(**a**) SEM image of the surface of the control group. (**b**) Surface view of the experiment groups. (**c**) Cross-sectional view of the experimental groups. (**d**) SEM images show osteoblast cells adhering to the scaffold surface on day 3 of the control, 13%ACP/CSH, and 15%ACP/CSH (left to right on second row) and the 18%ACP/CSH, 20%ACP/CSH, and 23%ACP/CSH (left to right on third row); yellow arrows indicate the osteoblast cells.

SEM revealed the obviously staggered filament morphology of the printed scaffolds of all experimental groups: 13%ACP/CSH, 15%ACP/CSH, 18%ACP/CSH, 20%ACP/CSH, and 23%ACP/CSH (Figure 2b,c). The control group showed that almost all filaments were fused (Figure 2a).

Osteoblast cell adhesion was seen in the SEM images on day 3. The experimental groups clearly expressed the characteristics of pseudopodia and body spreading on the top surface of the scaffold filaments while the control group showed a few round cells (Figure 2d).

### 3.2. Ion Complements and EDS-SEM Mapping

**Figure 3:**
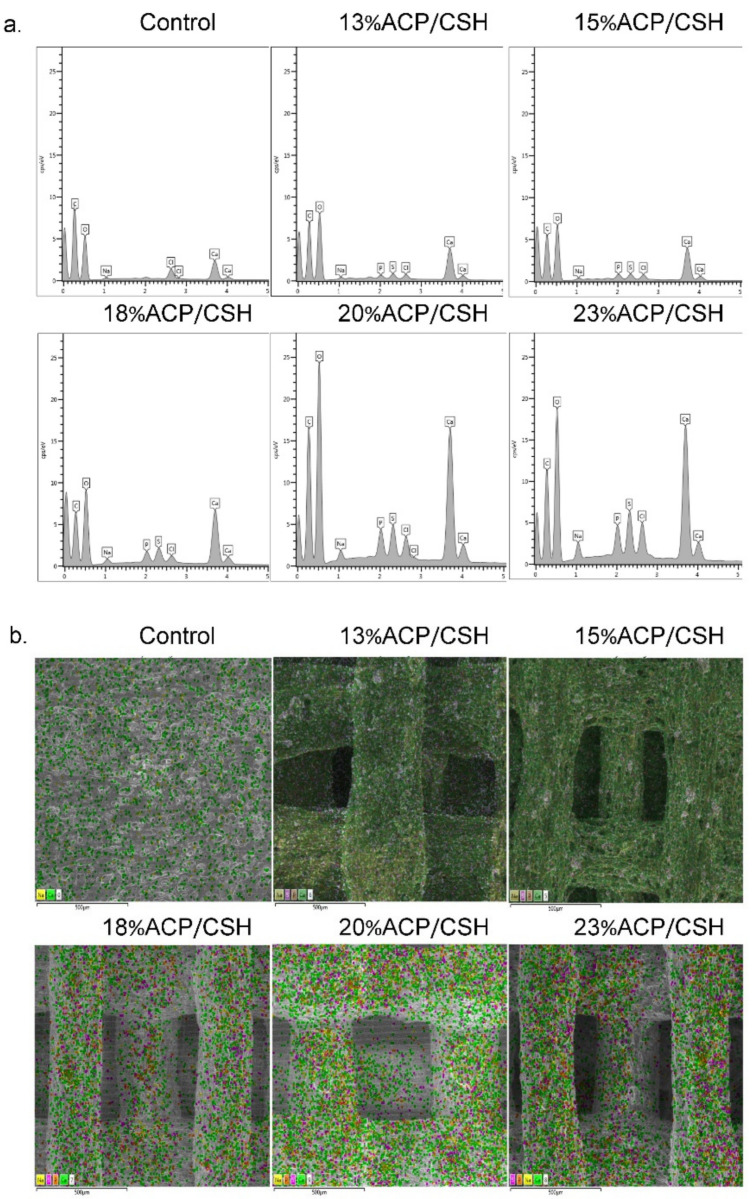
(**a**) Ion complement graphs show the amounts of C, O, Ca, P, Na, S, and Cl ions. (**b**) SEM mapping shows ion dispersion (green is Ca, orange is P, yellow is Na, pink is S, and red is Cl).

The EDS profile of all groups displayed the ion complements. About 40% of the ions were C and O. The Ca, P, Na, and S ions increased as the concentration of ACP/CSH increased. The Cl ions were shown to be <2.5% in all groups (Table 1 and Figure 3a).

Mapping of the SEM images revealed the distribution of each ion on the scaffolds. The pigment intensities of the Ca, P, Na, and S ions tended to increase from the control to the 23%ACP/CSH group. All ions displayed a homogenous dispersion on the filament areas (Figure 3b).

### 3.3. Calcium Release

**Figure 4:**
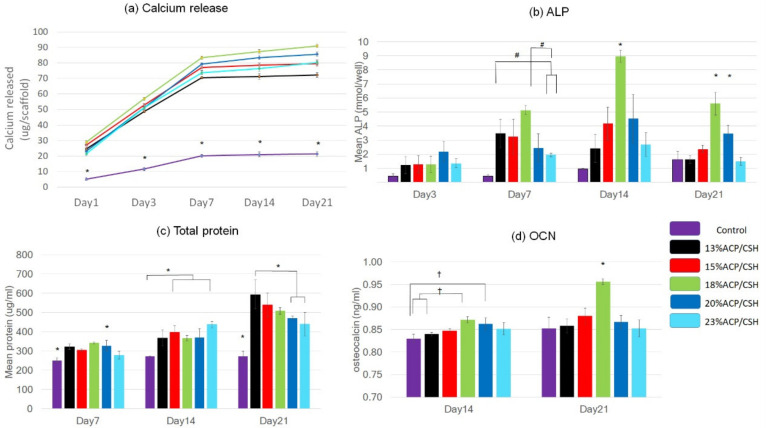
(**a**) Calcium release of the scaffolds soaked in PBS on days 1, 3, 7, 14, and 21. (**b**) ALP activity on days 3, 7, 14, and 21. (**c**) Total protein synthesis on days 7, 14, and 21. (**d**) Osteocalcin expression on days 14 and 21 (^†^ *p* < 0.05, ^#^ *p* < 0.01, * *p* < 0.001).

On day 1, the 18%ACP/CSH showed the highest calcium ion release while the control group revealed the lowest calcium ion release, with a significant difference (*p* < 0.001). On day 7, the 20%ACP/CSH group released significantly more calcium ions than the 13%ACP/CSH and 23%ACP/CSH groups. On day 3, all of the experiments found that the amount of calcium was not statistically different except only the control group (*p* < 0.001). We observed a trend in the calcium ion release, which decreased in all samples on days 14 and 21 (Figure 4a).

### 3.4. Alkaline Phosphatase (ALP) Analysis

The ALP analysis revealed that the 18%ACP/CSH group had the highest values on days 7, 14, and 21, which were significantly different from the others (*p* < 0.01, 0.001, 0.001 respectively). The 13%ACP/CSH and 15%ACP/CSH groups had the second highest levels, with higher ALP detected on day 7. However, on days 14 and 21, the 20%ACP/CSH group displayed remarkable measurements of ALP activity following the 18%ACP/CSH group (Figure 4b).

### 3.5. Total Protein Synthesis

On day 7, total protein was highly synthesized in the 13%ACP/CSH, 15%ACP/CSH, 18%ACP/CSH, and 20%ACP/CSH groups. The amount of protein increased until day 21, especially for the 13%ACP/CSH group, which showed the highest amount of protein synthesis, which was significantly different (*p* < 0.001) (Figure 4c).

### 3.6. Osteocalcin Assay (OCN)

The expression of OCN indicated mature osteoblast cells. The osteocalcin test revealed that the 18%ACP/CSH group significantly expressed the highest level of OCN (*p* < 0.05 on day 14, *p* < 0.001 on day 21) (Figure 4d).

### 3.7. Calcium Deposition and Mineralization

**Figure 5:**
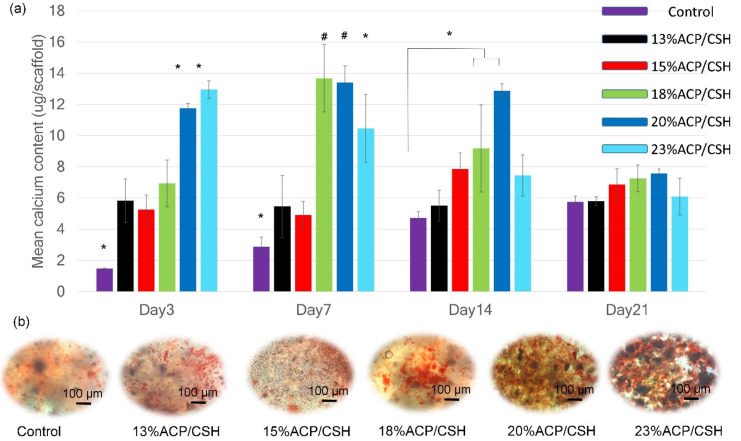
(**a**) Calcium deposition evaluated on days 3, 7, 14, and 21. (**b**) Calcium mineralization images represented by alizarin red stain on day 14 (^#^ *p* < 0.01, * *p* < 0.001).

Calcium deposition from the osteoblast cells on day 3 showed significantly higher levels in the 20%ACP/CSH and 23%ACP/CSH groups (*p* < 0.001) while the 18%ACP/CSH and 20%ACP/CSH groups provided the highest calcium deposition on day 7, with a significant difference (*p* < 0.01). However, on day 14, only the 20%ACP/CSH group continued to increase calcium deposition, which was statistically significant (*p* < 0.001). On day 21, calcium deposition decreased in all samples (Figure 5a).

The light microscopy images revealed calcium mineralization identified by alizarin red dye. On day 14, the 18%ACP/CSH and 20%ACP/CSH groups provided much denser granules, followed by the 23%ACP/CSH group (Figure 5b).

## 4. Discussion

Tissue engineering science has advanced greatly, and new bone tissue formation is necessary for repair of bone defects [23]. Chemical characteristics support osteoid formation due to inorganic deposits within the ECM, especially calcium and phosphate, which serve as important ions. No previous study has focused on the various amounts of calcium and phosphate compositions from a combination of ACP and CSH to fabricate tetra-polymers of bio-natural materials to fabricate scaffolds using 3D printing [24]. Furthermore, 3D-printed composite scaffolds can control the distribution and concentration of calcium and phosphate homogenously [25].

The 3D structure of the printed scaffolds showed suitable physical properties, including an interconnected porous structure, that mimic cancellous bone for cell attachment and penetration [26]. However, cell attachment and proliferation is just the initial step for cell growth [27] because cells need to differentiate into mature bone for new bone formation [28].

Osteoblast cell attachment and spreading were demonstrated in the SEM images on day 3. The control group demonstrated slow spreading of the cells due to the unstable structure of the Alg, which resulted in filopodia that were most likely diffused [29]. The experimental groups showed better spreading of the cells on the scaffold surface because of increased Ca ions from the ACP and CSH that could immediately react with the PO_4_^3^^−^ groups in the PBS buffer [30].

The amounts of Ca and P ions were similar to the initial powder concentrations in the study of Li and Weng [31], in which the EDS-SEM of ACP showed only Ca and P elements. However, in this study, the amount of Na ions was similar to the study by Franklin et al. [32] because the ACP powder was mixed in a PBS solution that likely contained Na ions. Therefore, it was hypothesized that a sodium-ion-rich surface could initially induce an apatite phase [32]. In addition, the Ca ions in the control group were available from the CaCl_2_ crosslinking agent as shown in the study by Mi et al. [25]. However, on day 14, the minimal amount of Ca ions had a low ability to promote bone marrow-derived mesenchymal stromal cells at a proliferative and differentiated rate. The highest Ca ion concentration of the 23%ACP/CSH group showed lower cell differentiation (ALP and OCN) than the 18%ACP/CSH group because the increased calcium content could slow osteopontin and bone sialoprotein gene expression. Therefore, the osteoblast cells exhibited delayed maturation [25].

The calcium release rate in this study demonstrated faster release on day 1 and a lower rate of release after day 14, which was the same as the study by Mi et al. In addition, the 20%ACP/CSH and 23%ACP/CSH groups had higher Ca and phosphate contents but a slow rate of calcium release because of the higher crystallinity [25]. Additionally, Kilian D et al. [33] reported that the Ca ions released from the 3D scaffold derived only from crosslinking agents had a lower Ca ion concentration than the culture medium, which was similar to the control group in this study. The ACP and CSH, on the other hand, can release higher Ca ions. Pei P. et al. [34] reported that 3D-printed 20–40% CSH allowed a Ca cumulative range of 50–60% on day 1, but our study used less CSH and obtained the same amount of released Ca.

The Ca and S ions from the CSH could initiate apatite formation because of the low pH, which can initiate an inflammatory process and exhibit osteogenic cell functions [35]. Although the S ions were released rapidly from the scaffold’s surface within 21 days, this study showed the highest ALP activity in the 18%ACP/CSH group on day 14. This occurred because during S ion release, the low pH could initiate the inflammatory process. This result was similar to the study by He et al., which found high ALP activity [35] that increased until days 14 on a 20–40% CSH 3D scaffold [34]. Furthermore, Ca and P could promote more immune and adipocyte cells and blood vessels up to week 8, which indicated that Ca-P deposition on the scaffold promoted mature bone formation [25]. High levels of protein synthesis and OCN expression were observed on days 14 and 21, especially in the 18%ACP/CSH group. This occurred because the amount of Ca and the remaining number of living cells probably started to adapt to the eco-environment for cell differentiation from the early stage to the late stage [30,35]. Furthermore, calcium deposition and alizarin red staining confirmed the numerous mineral nodules. In this study, the 18% ACP/CSH showed the highest ALP and OCN, but the mineralization in the 20% ACP/CSH group was noticeably higher than the 18% ACP/CSH group on day 14 because sulfate ions were rapidly released on day 1 and accumulated more in ECM until day 7, which may subsequently interact with bodily fluids and affect bone mineralization [34]. However, the mechanical properties of 23%ACP/CSH were less than 20%ACP/CSH, indicating that the scaffold with lower mechanical properties provides less cell differentiation [22].

## 5. Conclusions

The surface of the scaffolds fabricated in this research could promote ion deposition by the Ca-P-rich components. Here, 3D-printing is a new technology for fabricating scaffolds that promote homogenous dispersion of ions on all filaments along with a suitable pore size and interconnected porous structure. All experimental groups dispersed the Ca, P, Na, S, Cl, C, and O ions in the same percentages as their concentrations in the initial powder. The Ca ions in the experimental groups were highly released on days 1, 3, and 7 and subsequently decreased on days 14 and 21. The 18%ACP/CSH group showed the highest ALP activity on days 7, 14, and 21. All experimental groups exhibited protein synthesis that was not different at any time point. The 18%ACP/CSH group showed significantly high OCN expression on days 14 and 21. The 18%ACP/CSH, 20%ACP/CSH, and 23%ACP/CSH groups showed high levels of calcium deposition and mineralization. However, due to the limitations of murine cells, more in vitro experiments with human mesenchymal stem cells are needed before assessing bone regeneration in an animal study.

## Figures and Tables

**Table 1:** Weight ratios of the elements (C% + O% + Ca% + P% + Na% + S% + Cl% = 100%).

Groups	C		O		Ca		P		Na		S		Cl	
	Mean	SD	Mean	SD	Mean	SD	Mean	SD	Mean	SD	Mean	SD	Mean	SD
Control	50.21	1.57	41.88	1.38	5.65	0.27	0	0.00	0.41	0.06	0.00	0.00	1.86	0.12
13%ACP/CSH	38.49	0.47	49.86	0.69	8.74	0.34	0.63	0.02	0.51	0.06	0.84	0.08	0.94	0.09
15%ACP/CSH	38.30	0.54	49.25	0.40	9.25	0.64	0.84	0.13	0.52	0.03	0.88	0.21	0.95	0.06
18%ACP/CSH	37.02	1.63	47.53	0.75	10.26	0.43	1.23	0.15	0.74	0.05	1.49	0.17	1.73	1.05
20%ACP/CSH	37.18	1.49	46.82	1.26	10.23	0.57	1.44	0.10	1.10	0.07	1.95	0.07	1.28	0.17
23%ACP/CSH	34.14	0.55	45.60	0.14	12.38	0.37	1.83	0.12	1.52	0.08	2.20	0.42	2.31	0.15

## Data Availability

Not applicable.
